# Transtibial Femoral Tunnel Technique in ACL Reconstruction and Osteoarthritis Incidence

**DOI:** 10.1055/s-0044-1779328

**Published:** 2024-03-21

**Authors:** Marcus Vinicius Danieli, Allan Viktor Pires Molinari, João Vitor Guedes Suzze, Victoria de Abreu, João Paulo Fernandes Guerreiro

**Affiliations:** 1 Uniort.e – Hospital de Ortopedia, Londrina, PR, Brasil; 2 Pontifícia Universidade Católica PUC, Paraná, Campus de Londrina, Londrina, PR, Brasil

**Keywords:** anterior cruciate ligament reconstruction, anatomy, osteoarthritis, diagnostic imaging

## Abstract

**Objective** : Evaluate osteoarthritis incidence in patients that undergone ACL reconstruction using the transtibial technique, with a minimum of 5 years of follow up, with isolated ACL injury.

**Methods** : Patients who underwent ACL reconstruction by the same surgeon using the transtibial technique with hamstrings graft and with a minimum of 5 years of follow-up, without other injuries during the surgical procedure, were selected to undergo imaging exams of the operated knee to assess the incidence of osteoarthritis. The obtained data were evaluated by descriptive statistics.

**Results** : Forty-two patients (44 knees) were evaluated, with a mean age of 31 years old (SD: 8), being 23 right knees and 28 male patients. Mean time from surgery to imaging evaluation was 94.1 months (ranging from 60 to 154 months; SD: 28). Of the evaluated knees, 37 did not have osteoarthritis (83.3%) and 7 had (16.7%).

**Conclusion** : ACL reconstruction with femoral tunnel performed through the transtibial technique in patients without other associated injuries in the operated knee, using hamstrings graft, with a minimum of 5 years of follow up, showed an osteoarthritis incidence of 16.7% in a mean follow-up of 94.1 months.

Level Of Evidence V; Case Series.

## Introduction

The femoral tunnel position in Anterior Cruciate Ligament (ACL) reconstruction is a factor that could affect the knee biomechanics and kinematic. 1 The transtibial technique to perform this tunnel has been historically used, but it is questioned if this technique is really able to restore the anatomic position of the original ligament. 2 3 The incorrect placement of the tunnel could lead to instability, causing new injuries, accelerating the onset of osteoarthritis. 4

This led to the development of anatomical technique to perform the femoral tunnel. Literature shows this approach can result in more accurate graft positioning, higher knee stability and better functional results when compared to the transtibial technique. 5 6 7 8 9 10 However, another studies did not reach the same conclusion, with similar results between both techniques. 1 6 8 11

A recent meta-analysis showed that transtibial technique is associated to a higher knee osteoarthritis incidence after 5 year of follow up, but patients with meniscal or chondral injuries were not excluded, which is a great bias. 12 Another authors cite that the main factors associated to osteoarthritis after ACL injury and reconstruction surgery would be the original trauma intensity and the presence of associated meniscal or chondral injuries. 1 13 14 15

Thus, the objective of this study was to evaluate the knee osteoarthritis incidence in patients that underwent ACL reconstruction with the femoral tunnel performed by the transtibial technique, with a minimum of 5 years of follow up, without associated injuries to the knee at the day of surgery.

## Material and Methods

The study was approved by the Ethic and Research Committee of the institution, linked to the National Research Ethics Commission (CAAE 50743821.1.0000.5696). Patients from a private clinic who underwent ACL reconstruction by transtibial technique, with hamstrings graft, and with at least 5 years of follow-up, without any associated injury to the operated knee at the day of the surgery, were selected. All patients were operated on by the same surgeon. The patients were invited to perform X-Ray images of the operated knee to assess the presence of osteoarthritis.

All patients included in the study signed an informed consent form.

Patients were excluded if it was impossible to contact, to perform the images, declined to participated in the study, underwent ACL reconstruction revision or another ligament reconstruction of the affected knee, and meniscal or chondral surgery with more than 1 year of follow-up.

The accepted image exam was X-Ray (Orthostatic Anterior View; Rosenberg; and Lateral View). The Kellgren & Lawrence (KL) radiographic osteoarthritis classification was used. The grade I classification was already considered as the presence of osteoarthritis, corroborated by degenerative findings on magnetic resonance imaging. Only cases of KL grade I osteoarthritis were submitted to a resonance examination to confirm the presence of degenerative signs. The image exams were evaluated by two Orthopedic Surgeons.

The obtained results were analyzed by simple descriptive statistics, ie, obtaining the percentage of patients with osteoarthritis among those analyzed. Patients were also divided into 2 groups: between 5 and 10 of surgery, and with more than 10 years.

### Surgical Technique:

Patients were operated under spinal anesthesia. After adequate limb preparation a 3 cm longitudinal incision was made over the tibial hamstrings insertion. The semitendinous and gracilis tendons were harvested and prepared in a quadruple fashion. Then, a tourniquet was applied to the proximal thigh. Subsequently, arthroscopy was performed through standard portals to articular inspection and preparation for the ACL reconstruction.

**Fig. 1:**
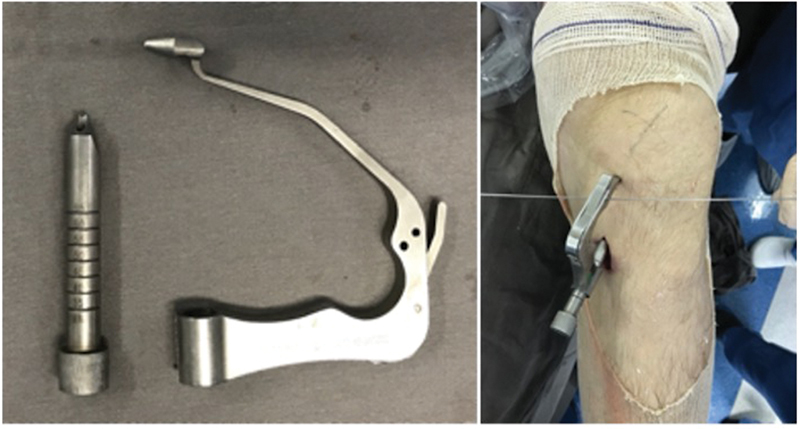
Left side: Howell's tibial guide. Right side: Using the guide in a left knee. Note K wire through the guide – this K wire must be parallel to the articular line to ensure the correct position.

**Fig. 2:**
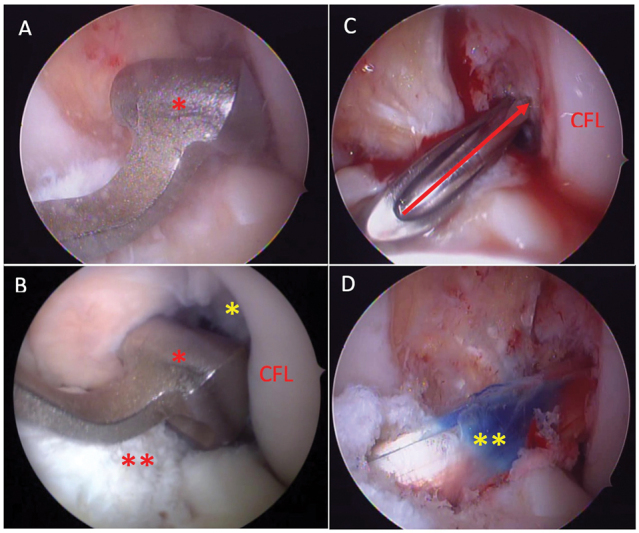
A: Howell's tibial guide being positioned (red asterisk). B: Extending the knee to lock the guide (red asterisk) in the femoral notch (yellow asterisk). Note the tibial remains of the ACL (double red asterisk). C: Transtibial guide positioning the guide wire (red arrow) at the femoral ACL insertion. D: Final view of the graft (double yellow asterisk). CFL: Lateral Femoral Condyle.

Howell's extension guide was used to create the tibial tunnel, applying a coronal plane inclination that allow the placement of the femoral tunnel at the anatomical ACL insertion. ( Figs. 1 and 2 ) After that, a guide pin is passed and a cannulated drill with the size of the graft is used through the guide. A femoral transtibial guide (bullseye) was used to place a guide pin, always checking if the guide is reaching the femoral anatomical ACL insertion. ( Fig. 2 ) Then, a endobutton drill (5mm diameter) is used through the guide until breaks the femoral cortex. Next, a drill with the same size of the tibial tunnel is used preserving the femoral cortex. The graft is then transposed into the tunnels and fixed with a titanium button to the femur and a titanium interference screw to the tibia.

The rehabilitation protocol was similar for all patients, with hospital discharge at the same day, allowing total weight bearing, with the help of crutches for gait safety for 7 days. Encouraged to start physical therapy immediately to range of motion gain and muscle activation. Evolution to light run is allowed with 3 months, specific sports movements starts with 5 months and return to pivoting sports between 7 to 9 months of follow-up.

The obtained data were evaluated by descriptive statistics.

## Results

**Fig. 3:**
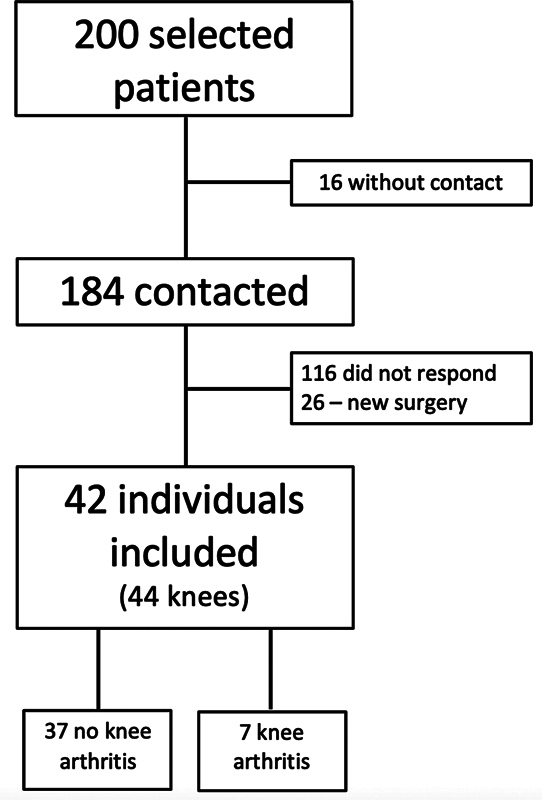
Study Flowchart.

Two hundred patients were selected, but 16 were excluded because of the lack of phone contact. Among the remaining 184, 116 did not answer the call and 26 underwent another surgery (1 due to a chondral injury, 10 for meniscal problems, 1 for Posterior Cruciate Ligament reconstruction and 14 required revision of the ACL reconstruction, all due to new trauma). A total of 42 individuals were included for analysis, and 2 had surgery on both knees at different times, resulting in evaluation of 44 knees ( Fig. 3 ).

There were 23 right knees, 28 male patients with mean age of 31 years old (SD: 8). Mean time from surgery until the imaging evaluation was 94.1 months (SD: 28) ( Table 1 ).

**Table 1:** Evaluated Patients Data

Right knee	23
Left knee	21
Male	28
Female	14
Age*	31 (16-46)
Time since surgery (months)*	94.1 (60-154)

*mean (minimum and maximum values).

Among the evaluated knees, there were 32 knees between 5 and 10 years of surgery with an osteoarthritis incidence of 13.51% (5 patients); and 7 with more than 10 years of surgery, with 71.4% (5 patients) without osteoarthritis ( Table 2 ).

**Table 2:** Osteoarthritis incidence in the evaluated knees

	Osteoarthritis	No Osteoarthritis
More than 10 years*	2 (28.6%)	5 (71.4%)
Between 5 and 10 years*	5 (13.51%%)	32 (86.49%)
Total	7 (16.7%)	37 (83.3%)

*between surgery and imaging.

Regarding the Kellgren & Lawrence classification, there were 1 knee classified as grade I, 3 grade II, 2 grade III and 1 grade IV ( Table 3 ).

**Table 3:** Kellgren & Lawrence classification between groups

	Grade I	Grade II	Grade III	Grade IV	Total
> 10 years	0	1	0	1	2
5-10 years	1	2	2	0	5
Total	1	3	2	1	7

## Discussion

The most important finding of the study is that the ACL reconstruction with the femoral tunnel performed through the transtibial technique in patients without associated injuries have low osteoarthritis incidence (16.7%) with a mean follow-up of 94.1 months.

These findings contradict the systematic review and meta-analysis of Cinque et al. 12 where the osteoarthritis incidence related to the transtibial technique was 49.3%. In the same study, the group between 5 to 10 years of follow-up presented a osteoarthritis incidence of 53.7%, being the group with the highest incidence of these diagnosis. The group of patients of our study between 5 to 10 years of follow-up presented an osteoarthritis incidence of only 13.51%. We believe that this difference happened because the study of Cinque et al. 12 does not adequately identify whether or not patients had associated injuries, which would be a crucial information. Literature shows that meniscectomy is more related to higher functional impairment and pain 13 15 and, along with chondral injuries, more osteoarthritis. 1 13 14 Franceschi et al. 8 in a retrospective study evaluated 88 patients with a minimum of 5 years of ACL reconstruction surgery, being 46 by transtibial technique and 42 anatomical, also excluding patients with meniscal and chondral injuries. They found similar results regarding function and evolution to degenerative changes for both techniques. This conclusion is in agreement with that obtained here.

It is known that the older the studies the greater the number of meniscectomies. Meniscal repair techniques have evolved and become more popular recently. Thus, it is assumed that the most recent studies may show a lower osteoarthritis incidence due to a higher meniscal preservation. 11 12 This suggests that the most important factor related to osteoarthritis in patients undergoing ACL reconstruction may not be the surgical technique but the presence or absence of associated injuries, mainly meniscal and chondral, and the choice of treatment of these injuries.

A differentiating factor in this study could be the use of the Howell's tibial guide. This guide create a tibial tunnel with greater inclination in the coronal plane that may allow to perform a anatomical femoral tunnel. The study by Cuzzolin et al. 11 mention that the crucial factor to be discussed is not how the femoral tunnel is made, but where it is made. Transtibial technique variations could allow to perform the femoral tunnel at the ACL anatomical insertion. This was demonstrated in the study by Piasecki et al. 16 where the authors used cadaver's knees with the help of navigation and image control with a C-arm. By testing different angles for the tibial entrance during performing the tibial tunnel, the authors showed it is possible to create anatomical femoral tunnels through the transtibial technique.

As study limitations the great loss of patients can be cited. The main causes were due to lack of contact or response and the use of only patients without other injuries than ACL. Even so, the number of evaluated individuals was very similar to other studies with similar objectives. 1 5 8 15 Another bias could be the use of Howell's ACL tibial guide, which is not widely adopted. However, the tunnel position can be replicated by using any standard ACL tibial guide, just by changing the guide pin inclination and tibial enter point. 16 The absence of a group using the anatomical technique to compare the results also weakens the power of this study. The inclusion of a group of patients using the same technique but with the presence of associated injuries, in order to compare the incidence of osteoarthritis, could also increase the power of the study. However it was decided to remove this factor and compare with the data already published in the literature. Patients activity degree, time between injury and surgery, knee stability and functional scores would be important because are factors that may be associated with the osteoarthritis development. However, these data were not available in the medical records for most of the individuals. The absence of radiographic images of the non-operated knee could also be a bias. Some patients could already show some signs of osteoarthritis regardless of the injury and surgery, and this comparison could show that.

## Conclusion

The ACL reconstruction performing the femoral tunnel through the transtibial technique in patients without another associated injuries to the operated knee, using quadruple hamstring graft, with a minimum of 5 years of follow up, showed an osteoarthritis incidence of 16.7% with a mean follow-up of 94.1 months.
