# Supplementary material for: Transtibial Femoral Tunnel Technique in ACL Reconstruction and Osteoarthritis Incidence
Source: Rev Bras Ortop (Sao Paulo). 2024 Mar 21;59(1):e76–81. doi: 10.1055/s-0044-1779328 (PMC10957279; doi:10.1055/s-0044-1779328)
Supplement: Related texts

# Research article: Técnica do túnel femoral transtibial na reconstrução do LCA e incidência de osteoartrite

**Keywords:** reconstrução do ligamento cruzado anterior, anatomia, osteoartrite, diagnóstico por imagem

## Abstract

**Objetivo** : Avaliar a incidência de osteoartrite em pacientes submetidos à reconstrução do LCA pela técnica transtibial, com seguimento mínimo de 5 anos, com lesão isolada do LCA.

**Métodos** : Pacientes que passaram por reconstrução LCA pelo mesmo cirurgião usando a técnica transtibial com enxerto de tendão dos músculos isquiotibiais e que foram acompanhados por no mínimo 5 anos, sem outras lesões durante o procedimento cirúrgico, foram selecionados para realizar exames de imagem do joelho operado a fim de avaliar a incidência de osteoartrite. Os dados obtidos foram avaliados por meio de estatísticas descritivas..

**Resultados** : Foram avaliados 42 pacientes (44 joelhos), com idade média de 31 anos (DP: 8), sendo 23 joelhos direitos e 28 pacientes do sexo masculino. O tempo médio entre a cirurgia e a avaliação por imagem foi de 94,1 meses (variando de 60 a 154 meses; DP: 28). Dos joelhos avaliados, 37 não apresentavam osteoartrite (83,3%) e 7 apresentavam (16,7%).

**Conclusão** : A reconstrução do LCA com túnel femoral realizado por meio da técnica transtibial em pacientes sem outras lesões associadas no joelho operado, utilizando enxerto dos tendões isquiotibiais, com um acompanhamento mínimo de 5 anos, apresentou uma incidência de osteoartrite de 16,7% em um acompanhamento médio de 94,1 meses.

Level of Evidence V; Case Series.

Nível de Evidência V; Série de casos

## Introdução

A posição do túnel femoral na reconstrução do Ligamento Cruzado Anterior (LCA) é um fator que pode afetar a biomecânica e a cinemática do joelho. 1 A técnica transtibial para a realização deste túnel tem sido historicamente utilizada, mas questiona-se se esta técnica é realmente capaz de restaurar a posição anatômica do ligamento original. 2 3 A colocação incorreta do túnel pode levar à instabilidade, ocasionando novas lesões, acelerando o aparecimento da osteoartrite. 4

Isso levou ao desenvolvimento da técnica anatômica para a realização do túnel femoral. A literatura mostra que esta abordagem pode resultar em posicionamento mais preciso do enxerto, maior estabilidade do joelho e melhores resultados funcionais quando comparada à técnica transtibial. 5 6 7 8 9 10 Porém, outros estudos não chegam à mesma conclusão, com resultados semelhantes entre as duas técnicas. 1 6 8 11

Uma metanálise recente mostrou que a técnica transtibial está associada a uma maior incidência de osteoartrite de joelho após 5 anos de acompanhamento, mas pacientes com lesões meniscais ou condrais não foram excluídos, o que é um grande viés. 12 Outros autores citam que os principais fatores associados à osteoartrite após lesão do LCA e cirurgia de reconstrução seriam a intensidade do trauma original e a presença de lesões meniscais ou condrais associadas. 1 13 14 15

Sendo assim, o objetivo deste estudo foi avaliar a incidência de osteoartrite de joelho em pacientes submetidos à reconstrução do LCA com túnel femoral realizado pela técnica transtibial, com acompanhamento mínimo de 5 anos, sem lesões associadas no joelho no dia da cirurgia.

## Material e métodos

O estudo foi aprovado pelo Comitê de Ética e Pesquisa da instituição, vinculado à Comissão Nacional de Ética em Pesquisa (CAAE 50743821.1.0000.5696). Foram selecionados pacientes de uma clínica privada submetidos à reconstrução do LCA pela técnica transtibial, com enxerto de isquiotibiais, e com pelo menos 5 anos de seguimento, sem qualquer lesão associada ao joelho operado no dia da cirurgia. Todos os pacientes foram operados pelo mesmo cirurgião. Os pacientes foram convidados a realizar radiografias do joelho operado para avaliar a presença de osteoartrite.

Todos os pacientes incluídos no estudo assinaram o termo de consentimento livre e esclarecido.

Foram excluídos os pacientes que tiveram impossibilidade de contato e/ou realização das imagens, recusaram participar do estudo, foram submetidos à revisão da reconstrução do LCA ou outra reconstrução ligamentar do joelho afetado e à cirurgia meniscal ou condral com mais de 1 ano de acompanhamento.

O exame de imagem realizado foi o Raio X (incidência antero-posterior; Rosenberg; e perfil). Foi utilizada a classificação radiográfica de osteoartrite de Kellgren & Lawrence (KL). A classificação grau I já foi considerada como presença de osteoartrite, corroborada por achados degenerativos na ressonância magnética. Apenas os casos de osteoartrite KL grau I foram submetidos a exame de ressonância para confirmar a presença de sinais degenerativos. Os exames de imagem foram avaliados por dois cirurgiões ortopédicos.

Os resultados obtidos foram analisados por estatística descritiva simples, ou seja, obtendo-se o percentual de pacientes com osteoartrite entre os analisados. Os pacientes também foram divididos em 2 grupos: entre 5 e 10 anos de cirurgia e com mais de 10 anos.

## Técnica Cirúrgica:

Os pacientes foram operados sob raquianestesia. Após preparação adequada do membro, foi feita uma incisão longitudinal de 3 cm sobre a inserção dos isquiotibiais na face medial da tibia proximal. Os tendões semitendíneo e grácil foram colhidos e preparados de forma quádrupla. Em seguida, foi aplicado um torniquete na região proximal da coxa. Posteriormente, foi realizada artroscopia através de portais padrão para inspeção articular e preparo para reconstrução do LCA.

O guia de extensão de Howell foi utilizado para criar o túnel tibial, aplicando uma inclinação do plano coronal que permite a colocação do túnel femoral na inserção anatômica do LCA. ( Figs. 1 e 2 ) Após isso, um pino-guia é inserido e uma broca canulada com o tamanho do enxerto é usada através do guia. Foi utilizado um guia femoral transtibial (bullseye) para posicionar o pino-guia, sempre verificando se o guia alcança a inserção anatômica femoral do LCA ( Fig. 2 ) Em seguida, uma broca para endobutton (5mm de diâmetro) é utilizada através do guia até romper a cortical femoral. Posteriormente, utiliza-se uma broca com o mesmo tamanho do túnel tibial preservando a cortical femoral. O enxerto é então transposto para os túneis e fixado com botão de titânio no fêmur e parafuso de interferência de titânio na tíbia.

**Fig. 1:** Lado esquerdo: guia tibial de Howell. Lado direito: Usando o guia no joelho esquerdo. Observe o fio K através do guia – este fio K deve estar paralelo à linha articular para garantir a posição correta.

**Fig. 2:** A: Guia tibial de Howell sendo posicionado (asterisco vermelho). B: Estendendo o joelho para travar o guia (asterisco vermelho) no intercôndilo femoral (asterisco amarelo). Observe os restos tibiais do LCA (asterisco vermelho duplo). C: Guia transtibial posicionando o fio-guia (seta vermelha) na inserção do LCA femoral. D: Vista final do enxerto (duplo asterisco amarelo). CFL: Côndilo Femoral Lateral.

O protocolo de reabilitação foi semelhante para todos os pacientes, com alta hospitalar no mesmo dia, permitindo carga total no membro operado, com o auxílio de muletas para segurança na marcha por 7 dias. Foi incentivado a iniciar fisioterapia imediatamente para ganho de amplitude de movimento e ativação muscular. A progressão para corrida leve foi permitida aos 3 meses, movimentos esportivos específicos começaram aos 5 meses, e o retorno a esportes com movimentos pivotantes ocorreu entre 7 e 9 meses de acompanhamento.

Os dados obtidos foram avaliados por estatística descritiva.

## Resultados

Foram selecionados 200 pacientes, mas 16 foram excluídos por falta de informações de contato. Dos 184 restantes, 116 não atenderam a ligação telefônica e 26 foram submetidos a outra cirurgia (1 por lesão condral, 10 por problema meniscal, 1 por reconstrução do ligamento cruzado posterior e 14 necessitaram de revisão da reconstrução do LCA, todos por novo trauma). Foram incluídos para análise 42 indivíduos, sendo que 2 foram operados em ambos os joelhos em momentos diferentes, resultando na avaliação de 44 joelhos ( Fig. 3 ).

**Fig. 3:** Fluxograma de estudo.

Foram 23 joelhos direitos, 28 pacientes do sexo masculino com idade média de 31 anos (DP: 8). O tempo médio desde a cirurgia até a avaliação por imagem foi de 94,1 meses (DP: 28) ( Tabela 1 ).

**Tabela 1:** Dados de pacientes avaliados

Joelho direito	23
Joelho esquerdo	21
Masculino	28
Feminino	14
Idade*	31 (16-46)
Tempo desde a cirurgia (meses)*	94.1 (60-154)

* média (valores mínimo e máximo).

Entre os joelhos avaliados, houve 32 joelhos entre 5 e 10 anos de cirurgia com incidência de osteoartrite de 13,51% (5 pacientes); e 7 com mais de 10 anos de cirurgia, sendo 71,4% (5 pacientes) sem osteoartrose ( Tabela 2 ).

**Tabela 2:** Incidência de osteoartrite nos joelhos avaliados

	Osteoartrite	Sem osteoartrite
Mais de 10 anos*	2 (28.6%)	5 (71.4%)
Entre 5 e 10 anos*	5 (13.51%%)	32 (86.49%)
Total	7 (16.7%)	37 (83.3%)

*entre cirurgia e exames de imagem.

Quanto à classificação de Kellgren & Lawrence, houve 1 joelho classificado como grau I, 3 grau II, 2 grau III e 1 grau IV ( Tabela 3 ).

**Tabela 3:** Classificação de Kellgren & Lawrence entre grupos

	Grau I	Grau II	Grau III	Grau IV	Total
> 10 anos	0	1	0	1	2
5-10 anos	1	2	2	0	5
Total	1	3	2	1	7

## Discussão

O achado mais importante do estudo é que a reconstrução do LCA com túnel femoral realizada pela técnica transtibial em pacientes sem lesões associadas apresenta baixa incidência de osteoartrite (16,7%) com seguimento médio de 94,1 meses.

Esses achados contradizem a revisão sistemática e meta-análise de Cinque et al. 12 onde a incidência de osteoartrite relacionada à técnica transtibial foi de 49,3%. No mesmo estudo, o grupo entre 5 e 10 anos de acompanhamento apresentou incidência de osteoartrite de 53,7%, sendo o grupo com maior incidência desses diagnósticos. O grupo de pacientes do nosso estudo entre 5 e 10 anos de seguimento apresentou incidência de osteoartrite de apenas 13,51%. Acreditamos que essa diferença ocorreu porque o estudo de Cinque et al. 12 não identifica adequadamente se os pacientes apresentavam lesões associadas ou não, o que seria uma informação crucial. A literatura mostra que a meniscectomia está mais relacionada a maior comprometimento funcional e dor 13 15 e, junto com lesões condrais, mais osteoartrite. 1 13 14 Franceschi et al. 8 em estudo retrospectivo avaliaram 88 pacientes com no mínimo 5 anos de cirurgia de reconstrução do LCA, sendo 46 pela técnica transtibial e 42 anatômicas, excluindo também pacientes com lesões meniscais e condrais. Eles encontraram resultados semelhantes em relação à função e evolução das alterações degenerativas para ambas as técnicas. Esta conclusão está de acordo com a obtida aqui.

Sabe-se que quanto mais antigos os estudos, maior o número de meniscectomias. As técnicas de reparo meniscal evoluíram e se tornaram mais populares recentemente. Assim, supõe-se que os estudos mais recentes possam mostrar uma menor incidência de osteoartrite devido a uma maior preservação meniscal. 11 12 Isto sugere que o fator mais importante relacionado à osteoartrite em pacientes submetidos à reconstrução do LCA pode não ser a técnica cirúrgica, mas a presença ou ausência de lesões associadas, principalmente meniscais e condrais, e a escolha do tratamento dessas lesões.

Um fator diferenciador neste estudo poderia ser a utilização do guia tibial de Howell. Este guia cria um túnel tibial com maior inclinação no plano coronal que pode permitir a realização de um túnel femoral anatômico. O estudo de Cuzzolin et al. 11 menciona que o fator crucial a ser discutido não é como é feito o túnel femoral, mas onde ele é feito. Variações da técnica transtibial poderiam permitir a realização do túnel femoral na inserção anatômica do LCA. Isso foi demonstrado no estudo de Piasecki et al. 16 onde os autores utilizaram joelhos de cadáveres com o auxílio de navegação e controle de imagem com um braço em C. Ao testar diferentes ângulos para a entrada tibial durante a realização do túnel tibial, os autores mostraram que é possível criar túneis femorais anatômicos por meio da técnica transtibial.

Como limitações do estudo pode-se citar a ausência de muitos pacientes. As principais causas foram por falta de informação de contato ou resposta e utilização apenas de pacientes sem outras lesões além do LCA. Mesmo assim, o número de indivíduos avaliados foi muito semelhante a outros estudos com objetivos semelhantes. 1 5 8 15 Outro viés poderia ser o uso do guia tibial do LCA de Howell, que não é amplamente adotado. Entretanto, a posição do túnel pode ser replicada usando qualquer guia tibial padrão do LCA, apenas alterando a inclinação do pino-guia e o ponto de entrada tibial. 16 A ausência de um grupo utilizando a técnica anatômica para comparar os resultados também enfraquece o poder deste estudo. A inclusão de um grupo de pacientes utilizando a mesma técnica, mas com presença de lesões associadas, para comparar a incidência de osteoartrite, também poderia aumentar o poder do estudo. Contudo optou-se por retirar este fator e comparar com os dados já publicados na literatura. O grau de atividade do paciente, o tempo entre a lesão e a cirurgia, a estabilidade do joelho e os escores funcionais seriam importantes porque são fatores que podem estar associados ao desenvolvimento da osteoartrite. Entretanto, esses dados não estavam disponíveis nos prontuários da maioria dos indivíduos. A ausência de imagens radiográficas do joelho não operado também poderia ser um viés. Alguns pacientes já podem apresentar alguns sinais de osteoartrite, independentemente da lesão e da cirurgia, e esta comparação poderia evidenciar este fato.

## Conclusão

A reconstrução do LCA realizando o túnel femoral pela técnica transtibial em pacientes sem outras lesões associadas no joelho operado, utilizando enxerto quádruplo de isquiotibiais, com acompanhamento mínimo de 5 anos, mostrou incidência de osteoartrite de 16,7% com seguimento médio de 94,1 meses.
